# Linking Inflammatory Bowel Disease Symptoms to Changes in the Gut Microbiome Structure and Function

**DOI:** 10.3389/fmicb.2021.673632

**Published:** 2021-07-19

**Authors:** Sayf Al-Deen Hassouneh, Mark Loftus, Shibu Yooseph

**Affiliations:** ^1^Burnett School of Biomedical Sciences, Genomics and Bioinformatics Cluster, University of Central Florida, Orlando, FL, United States; ^2^Department of Computer Science, Genomics and Bioinformatics Cluster, University of Central Florida, Orlando, FL, United States

**Keywords:** microbiome, gut, IBD, species, shotgun sequencing, association, networks

## Abstract

Inflammatory bowel disease (IBD) is a chronic disease of the gastrointestinal tract that is often characterized by abdominal pain, rectal bleeding, inflammation, and weight loss. Many studies have posited that the gut microbiome may play an integral role in the onset and exacerbation of IBD. Here, we present a novel computational analysis of a previously published IBD dataset. This dataset consists of shotgun sequence data generated from fecal samples collected from individuals with IBD and an internal control group. Utilizing multiple external controls, together with appropriate techniques to handle the compositionality aspect of sequence data, our computational framework can identify and corroborate differences in the taxonomic profiles, bacterial association networks, and functional capacity within the IBD gut microbiome. Our analysis identified 42 bacterial species that are differentially abundant between IBD and every control group (one internal control and two external controls) with at least a twofold difference. Of the 42 species, 34 were significantly elevated in IBD, relative to every other control. These 34 species were still present in the control groups and appear to play important roles, according to network centrality and degree, in all bacterial association networks. Many of the species elevated in IBD have been implicated in modulating the immune response, mucin degradation, antibiotic resistance, and inflammation. We also identified elevated relative abundances of protein families related to signal transduction, sporulation and germination, and polysaccharide degradation as well as decreased relative abundance of protein families related to menaquinone and ubiquinone biosynthesis. Finally, we identified differences in functional capacities between IBD and healthy controls, and subsequently linked the changes in the functional capacity to previously published clinical research and to symptoms that commonly occur in IBD.

## Introduction

Inflammatory bowel disease (IBD) is a heterogeneous disorder characterized by chronic inflammation of the gastrointestinal tract. The two main manifestations of IBD are Crohn’s Disease (CD) and Ulcerative Colitis (UC). CD most often affects the terminal ileum but can affect any part of the gastrointestinal tract in a non-contiguous fashion, sometimes known as ‘skip lesions,’ and often results in diarrhea, bloody stools, abdominal pain, cachexia, and fatigue (Flores et al., 2015; Veauthier and Hornecker, 2018). UC most often affects the large intestine, extending from the rectum, and is characterized by contiguous inflammation and often results in rectal bleeding, bloody stools, diarrhea, cachexia, and fatigue (Flores et al., 2015; Yu and Rodriguez, 2017). While the etiology of IBD is not well understood, it is believed that the disorder arises due to environmental and host-related factors causing an aberrant immune response in genetically predisposed individuals (Kish et al., 2013; Chiara et al., 2020). One such factor is believed to be the microbiome, specifically the gut microbiome (Duranti et al., 2016).

The human microbiome is the collection of microbes that exists on and within the human body, and this collective has been implicated in maintaining health, as well as possibly contributing to a multitude of diseases such as IBD, Irritable Bowel Syndrome (IBS), diabetes, Parkinson’s disease, and amyotrophic lateral sclerosis (Brown et al., 2011; Gevers et al., 2014; Wu et al., 2015; Petrov et al., 2017; Kho and Lal, 2018; Vich Vila et al., 2018). The bacterial composition of the microbiome can be studied using DNA sequencing, either by targeted sequencing of a marker gene or by shotgun sequencing. Targeted sequencing involves the amplification of specific regions of bacterial genomes, such as the 16S ribosomal RNA gene, for use as a phylogenetic marker (Fox et al., 1977). However, due to the highly conserved nature of the marker genes, such as 16S rRNA gene, and the short lengths of the regions within the gene that are commonly targeted, the taxonomic resolution generated by these types of studies are often inadequate to distinguish bacterial species and accurate relative abundance estimation is difficult (Fox et al., 1992; Rastogi et al., 2009; Ranjan et al., 2016; Ibal et al., 2019). In contrast, shotgun sequence data generated from the DNA extracted from a sample can be used to obtain more accurate estimates of relative abundance, higher resolution of bacterial taxonomy, and a more accurate representation of genomic functional capacity (Ranjan et al., 2016; Laudadio et al., 2018).

Regardless of the sequencing framework used, the generated sequence data are compositional in nature, in that it is only possible to infer *relative* abundances of the constituent microbial taxa from these data (and not *absolute* abundances) (Gloor et al., 2017). This compositionality aspect makes it difficult to analyze differential abundance, infer associations, and estimate correlations (Aitchison, 1982; Pearson, 1896; Friedman and Alm, 2012; Tsilimigras and Fodor, 2016). By utilizing a Centered Log-Ratio (CLR) transformation of the relative abundance data, we can examine the differential abundances more clearly and without inducing spurious correlations (Aitchison, 1982; Pearson, 1896; Friedman and Alm, 2012; Tsilimigras and Fodor, 2016). Furthermore, the covariance matrix of log-transformed relative abundance data provides a good approximation of the covariance matrix of the log-transformed absolute abundance data enabling us to better model the associations between bacteria (Kurtz et al., 2015).

Associations within a bacterial community are comprised of the direct and indirect interactions between the community constituents and are important for understanding the underlying dynamics at play in a microbial community (Kurtz et al., 2015). Bacterial association networks are often constructed using pairwise correlation methods on relative abundance or count data of the bacteria found within the samples. Due to the compositional nature of sequencing data, however, it is difficult to accurately identify correlations from counts generated from sequencing data due to spurious correlations that arise (Friedman and Alm, 2012). Even after CLR-transformation of the sequencing data, pairwise correlation methods are unable to account for conditional independence between bacterial species causing these methods to produce inaccurate bacterial association networks (Kurtz et al., 2015). In this paper, we used a Gaussian Graphical Model (GGM) framework in conjunction with a graphical lasso (glasso) to construct bacterial association networks from the CLR-transformed relative abundance data (Friedman et al., 2008; Loftus et al., 2021). Utilizing the GGM framework on the CLR-transformed data, enables us to approximate the covariance structure of the absolute abundances as well as account for conditional independence between the constituent species (Aitchison, 1982; Wermuth and Lauritzen, 1990).

Due to the Random Forest Classifier’s (RFC) ability to deal with ‘noisy,’ non-normally distributed, multi-dimensional data, it has become an important tool for identifying important features and differences in the microbiome (Breiman, 2001; Shi et al., 2005; Díaz-Uriarte and Alvarez de Andrés, 2006; Saulnier et al., 2011; Loomba et al., 2017; Roguet et al., 2018). These features can include bacterial relative abundances and metadata thus allowing us to generate a model that accounts for subject characteristics as well as gut microbiota taxonomic profiles. Another benefit of the RFC is its ability to assign importance to the features used for the classification. The feature importance’s allow us to quantify the role a specific feature plays in making a prediction and can allow us to determine which features may be informative. One shortcoming of these feature importance’s, however, is their lack of statistical significance. Due to the stochastic nature of model construction using an RFC, some features may be relatively important in one instance of an RFC modeled using a specific diagnosis label, but relatively unimportant in another instance of the RFC modeled using the same diagnosis label as the previous model. To enable us to utilize RFC feature importance to distinguish potentially important features and reduce the dimensionality of our data, we formulated a framework that allowed us to add statistical significance to the feature importance’s.

Here, we utilized the IBD Multi-omics DataBase (IBDMDB) cohort from a previously published study to study IBD (Lloyd-Price et al., 2019). This dataset consists of shotgun sequence data generated from CD, UC, and an internal control group (henceforth also referred to as non-IBD samples). The non-IBD samples were collected from subjects that underwent histopathologic examination (via colonoscopy) but were not diagnosed with IBD. These samples are derived from subjects presenting for routine screenings, gastrointestinal (GI) distress, or non-specific symptoms generating a heterogeneous control group. This control group design may obfuscate important differences between healthy and IBD gut microbiomes, especially if the differences may be related to presentations common between IBD and GI distress, such as diarrhea, bloating, or abdominal pain. Additionally, many studies examining the microbiome suffer from a lack of cross-cohort consistency making it difficult to generalize findings to populations rather than just the utilized study groups (Pasolli et al., 2016). One proposed remedy for this lack of cross-cohort consistency is to utilize external samples from independent cohorts, especially when comparing diseased and healthy microbiomes, and applying the same methods and techniques across all samples (Pasolli et al., 2016; Thomas et al., 2019). To enable us to generalize our findings and utilize healthy control groups in our analysis, we incorporated samples from both the Human Microbiome Project (Huttenhower et al., 2012) referred to as the Healthy-1 cohort, and from Johnson et al. (2019) referred to as the Healthy-2 cohort, as external controls. The external cohorts we elected to use were shotgun sequence datasets generated from fecal samples collected from healthy subjects (no overt or reported disease) and utilizing the same sequencing platform as the IBDMDB cohort (Illumina). Furthermore, due to the similarity of the results produced by the Chemagic DNA extraction kit (IBDMDB cohort) and the Mo Bio PowerSoil DNA extraction kit (Healthy-1 and Healthy-2 cohorts), we concluded that these cohorts could serve as external controls without the addition of a significant amount of technical bias (Multinu et al., 2018). Also, due to the use of replicates within the Healthy-2 cohort and the IBDMDB cohort, we were able to examine temporal variation within subjects diagnosed with IBD relative to the non-IBD group (internal control) and the Healthy-2 group (external control). By incorporating these two independent healthy cohorts, we can compare the IBD samples to healthy samples and mitigate the possible issues inherent in the design of the IBDMDB internal control group (non-IBD group) as well as arrive at more robust and generalizable conclusions from our analysis.

To understand the effects of changes in the microbiome, we cannot solely focus on the presence, absence, or differential abundances that are found. We also need to examine the bacterial associations as well as the functional differences to understand how the microbiome is being shaped (Heintz-Buschart and Wilmes, 2018). By examining the taxonomy, the bacterial associations, and the functional changes of the gut microbiome, our study aims to identify bacterial species that may play a role in the onset or exacerbation of IBD or IBD-related symptoms. By utilizing two external healthy controls, we are also able to corroborate our conclusions when comparing IBD and healthy samples and generalize our findings more confidently to the population. Additionally, we utilized a machine learning framework and a prevalence threshold to identify potentially important bacterial species. We also compared the functional capacity of the gut microbiome of IBD samples to non-IBD and control samples and identified important potential functional differences that may play a role in symptoms IBD patients typically experience.

## Materials and Methods

### Data Acquisition

Shotgun sequence data generated from 574 fecal samples were obtained from three previously published studies of the human gut microbiome (United States populations). Of these, two cohorts were downloaded from NCBI’s Sequence Read Archive (SRA): Human Microbiome Project (SRA: PRJNA48479; 203 samples) and the IBD Multi-omics Database (SRA: PRJNA398089; 257 samples). The Johnson et al. (2019) cohort was downloaded from the European Nucleotide Archive (ENA) (ENA: PRJEB29065; 114 samples). We were able to access metadata for sex and age/age-group for all cohorts.

### Data Pre-processing

Reads from the whole genome sequencing data were trimmed using Trimmomatic (version 0.36) and then reads corresponding to the human genome were filtered out using BowTie2 (version 5.4.0) and the GRCh38.p12^1^ human reference genome (Langmead and Salzberg, 2012; Bolger et al., 2014).

### Read Mapping and Taxonomic Identification

Reads from each sample were mapped to 10,839 bacterial reference strain genomes obtained from the NCBI RefSeq database using BowTie2 (O’Leary et al., 2016). Bacterial genome relative abundances in each sample were estimated using a probabilistic framework based on a mixture model. The framework utilized an Expectation-Maximization (EM) algorithm to perform soft assignment of the reads to the reference genomes and was found to be highly accurate (Xia et al., 2011; Loftus et al., 2021). We have previously demonstrated that samples with less than 250,000 mapped reads display diminished accuracy for taxonomic profiling, consequently all samples that contained less than 250,000 mapped reads threshold were not used for downstream analysis (Loftus et al., 2021). When calculating relative abundances, any value below 1 × 10^–5^ was considered statistical noise (and set to 0). For each sample, the relative abundances of strains belonging to the same species were aggregated. In this manner, an *n* x *D* sample-taxa matrix was created from *n* input samples and *D* species. Entry (*i,j*) in this matrix represents the relative abundance of species *j* in sample *i*. Row *i* is also referred to as the sample relative abundance vector for sample *i*, and the values in this vector sum to 1. The sample relative abundance vectors were then transformed using the CLR transformation and the CLR-transformed data was used for all downstream analyses except for the alpha-diversity analysis. The CLR transformation for a vector *x* (i.e., row of sample-taxa matrix) is defined as:

$$C\,L\,R\,\left(x\right)=\left[l\,n\,\frac{x_{1}}{G\,\left(x\right)},l\,n\,\frac{x_{2}}{G\,\left(x\right)}\,\ldots \,l\,n\,\frac{x_{D}}{G\,\left(x\right)}\right]$$

where *x* is the sample relative abundance vector, *D* is the total number of species, and *G(x)* is the geometric mean of *x*. The geometric mean is defined as:

$$G\,\left(x\right)=\sqrt[{D}]{x_{1}\times x_{2}\times \ldots \,x_{D}}.$$

### Sample Inclusion Criteria

#### IBDMDB Inclusion Criteria

To reduce potential confounders within the internal control group (non-IBD samples), we instituted a set of inclusion criteria for the non-IBD group: no colonoscopy within the last 2 weeks, no history of bowel surgery, no immunosuppressants use, no antibiotic use, no IBS, and no diarrhea in the past 2 weeks. Due to the adverse associations between these variables and the gut microbiome that have been noted in the literature, we excluded any samples from subjects that violated these criteria (Dethlefsen et al., 2008; Schubert et al., 2014; Bhat et al., 2017; Halfvarson et al., 2017; Vich Vila et al., 2018; Nagata et al., 2019). We also did not utilize any samples collected prior to week 26 of the study to ensure that subjects had ample time to overcome any gastrointestinal distress they have been experiencing at the time of study initiation. To limit any potential bias from an over-representation of a subject within the cohort, no more than five randomly chosen samples were retained from any one subject for any of the sample groups in the IBDMDB cohort (CD, UC, non-IBD) resulting in a mean number of replicates of 2.5 and a median of 2.

#### Healthy-1 Cohort Inclusion Criteria

Samples for the healthy-1 cohort were derived from Huttenhower et al. (2012) and were generated as part of the Human Microbiome Project. All 203 samples utilized were derived from unique individuals and demonstrated over 250,000 mapped reads so all samples were included in the analysis.

#### Healthy-2 Cohort Inclusion Criteria

Samples for the healthy-2 cohort were derived from Johnson et al. (2019) and were generated as part of a longitudinal analysis of fecal shotgun metagenomes in healthy subjects. The study by Johnson et al. (2019) aimed to examine gut microbiome responses to a changes diet. Subject were randomly given fatty acid supplementation on days 10–17 of the study. To ensure that our analysis reflected healthy samples on habitual diets, only samples taken prior to day 10 of the study were used. Furthermore, subjects were sampled daily for 17 days but not all subjects consistently had more than five samples with greater than 250,000 (minimum threshold for inclusion) mapped reads so to limit the number of replicates from a single subject a maximum of five randomly chosen samples were retained from any one subject resulting in a mean number of replicates of 3.3 and a median of 3.

### Diversity Analysis

Alpha diversity was analyzed using the Shannon entropy. The Shannon entropy, H, is defined as:

H=-∑i=1Dpilog(pi)2

where *D* is the number of species in the sample and *p*_*i*_ is the proportion of species *i* in the sample (Shannon, 1948). The non-transformed relative abundances were used for the Shannon entropy calculations.

### Intrapersonal and Interpersonal Dissimilarity

The Bray–Curtis dissimilarity (BCD) between replicates within a subject was used to quantify intrapersonal variation within each cohort with replicates (IBDMDB and Healthy-2 cohorts). The BCD between subjects within diagnosis groups (interpersonal dissimilarity) was also examined to observe the variability of the gut microbiota within the diagnosis groups. The BCD between two samples, *i* and *j*, was calculated as

$$B\,C\,D_{i,j}=1-\frac{2\,C_{i\,j}}{S_{i}+S_{j}}$$

where C_*ij*_ is the sum of the relative abundances of the species with the lowest combined relative abundance within samples *i and j.* S_*i*_ and S_*j*_ are the sums of the relative abundances found in sample *i* and sample *j*, respectively. The intrapersonal dissimilarity was calculated by generating pairwise BCD’s for samples from the same subject. The interpersonal dissimilarity was calculated by generating pairwise BCD’s between samples from different subjects.

### Prevalent Species

To reduce the dimensionality of our data, we utilized only bacterial species that were present in at least 90% of samples within each diagnosis group (IBD, non-IBD, Healthy-1, and Healthy-2) for our downstream analysis (Loftus et al., 2021). The union of the bacterial species present at a prevalence greater than or equal to 90% in each diagnosis group was then used for the classification of the signature species.

### Classification of Signature Species

A modified Random Forest Classifier (RFC) framework was used to identify bacterial species for downstream analysis (Breiman, 2001). The RFC was used to classify samples by the sample groups (IBD, non-IBD, and Healthy). The Healthy-1 and Healthy-2 cohort were combined for the RFC analysis to enable us to identify bacterial species importance’s by health status, rather than by cohort. A random noise column was added into the data prior to RFC analysis. The noise column was generated by creating a normal distribution resembling the CLR-transformed data of the genome relative abundances and randomly sampling from the distribution. The data was then label encoded due to the presence of categorical data. This process was performed 100 times, where a new random noise column would be generated each time, and the feature importance’s of every feature (bacterial species, metadata, and the random feature) were stored for all runs. A Mann–Whitney *U* test (Mann and Whitney, 1947) was then performed on the importance’s of all features with a mean feature importance higher than the random feature to determine if the importance’s of these features were significantly different from the feature importance’s of the random column. The Benjamini–Hochberg procedure for controlling false discovery rate was utilized to account for the multiple-testing and only features with a *q*-values less than 0.05 were considered significantly different from the random column (Benjamini and Hochberg, 1995). This framework allows us to identify the bacterial species and metadata whose feature importance’s were significantly higher than the random noise. The bacterial species that were significantly more important than the random noise column are referred to as the ‘signature’ species due to their ability to provide a non-random signal during classification. The RFC was implemented in Python 3.8 using Sci-kit Learn 0.23.1 (Van Rossum and Drake, 2009; Varoquaux et al., 2015).

#### Cross-Validation of Random Forest Classifier

To estimate RFC model accuracies, we utilize a train-test sample split of 70% for training and 30% for testing. The testing data was then used to estimate the accuracy of the RFC model using the F1-score metric. The F1-score, also known as the harmonic mean of precision and recall, is defined as

$$F\,1=\frac{2\,\left(p\times r\right)}{p+r}$$

Where *p* is defined as precision and *r* is defined as recall.

### Differential Abundance Analysis

Differential abundance analysis was conducted by performing a Mann–Whitney *U* test and the Benjamini–Hochberg multi-test correction on the CLR-transformed relative abundance profiles. The IBD group was compared to the non-IBD group, the Healthy-1 group, and the Healthy-2 group individually. Bacterial species that were significantly differentially abundant in IBD relative to every other individual group were designated as differentially abundant.

### Bacterial Association Network Construction

We represent bacterial association networks using an unweighted graph in which nodes denote bacterial species and an edge between two nodes denotes an association between the corresponding bacterial species. The signature species were used to create a sample-taxa matrix of CLR-transformed relative abundances in each sample. The GGM framework, as previously described, was used to generate the bacterial association networks using the above sample-taxa matrices for each cohort (Loftus et al., 2021). In brief, the HUGE package in R was used to compute a sparse precision matrix. The stability approach to regularization selection (StARS) method was used to determine the tuning parameter in the l_1_-penalty model for sparse precision matrix estimation. To reduce false positives, the final precision matrix, Ω, underwent bootstrap testing. If Ω[i,j] ≠ 0, then Ω’[i,j] = Ω[i,j] if[*i*,*j*]≠0 in *f^∗^r* or greater precision matrices estimated from bootstraping. Otherwise, Ω’[i,j] = 0. The value *r* = 50 (bootstrap replicates) and *f* = 0.8 (threshold between 0 and 1 indicating proportion of edges that must be non-zero). Networks were visualized and analyzed using Python 3.8 and NetworkX 2.4 (Hagberg et al., 2008).

### Eigenvector Centrality

Eigenvector centrality (EVC) measures the influence a node has in a network by accounting for the connections of the node in question as well as the connections of its neighbors (Bonacich, 1972; Ruhnau, 2000). The EVC, *x*, for a given node, *i*, is defined as:

$$x_{i}=\sum_{j}A_{i\,j}\,x_{j}$$

where *A* is the adjacency matrix and *j* is a neighboring node of *i*.

### Bacterial Genome Functional Annotation

Prodigal (version 2.6.3) was used to identify genes and generate protein sequence translations (Hyatt et al., 2010). The protein sequence translations were provided to InterProScan (version 5.39-77.0) to identify protein families using the TIGRFAM (versions 15.0) protein family database (Haft, 2001; Hunter et al., 2009). TIGRFAM counts were generated for each reference genome. Bacterial species that were greater than 90% prevalent within a diagnosis group (IBD, non-IBD, Healthy-1, and Healthy-2) were used for functional annotation to reduce the effects of potentially transient species when analyzing the genomic functional capacity of the microbiomes (Ursell et al., 2012; Saunders et al., 2016). Then the TIGRFAM counts were weighted based on CLR-transformed genome relative abundance and summed by total for each cohort. Differential abundances of TIGRFAM profiles were therefore calculated by using the CLR-transformed relative abundances of the TIGRFAMs within each cohort. The TIGRFAM CLR-transformed relative abundances were then tested using a Mann–Whitney *U* test.

### Statistical Analysis and Graph Creation

Statistical analysis and graph creation was performed using Python 3.8 (Van Rossum and Drake, 2009).

## Results

A total of 574 shotgun sequence datasets from 3 previously published studies (IBDMDB, Healthy-1, and Healthy-2) of the human gut microbiome were utilized in this study. The IBDMDB cohort consisted of CD, UC, and non-IBD samples. To minimize potential confounders in the IBDMDB group, samples from individuals that reported recent colonoscopy, antibiotic or immunosuppressant use, IBS, or recent GI symptoms were excluded from the control (non-IBD) group. For each dataset, the sequence reads were quality trimmed and human reads were identified and filtered. The remaining reads were mapped to a comprehensive collection of 10,839 bacterial strain reference genomes from NCBI RefSeq and genome relative abundances were calculated using a probabilistic framework (Xia et al., 2011; Loftus et al., 2021). The alpha-diversity was then calculated on the relative abundances using Shannon entropy. To reduce the dimensionality of our data, we focused our analysis on bacterial species that were prevalent in at least 90% of the samples. Next, the sample relative abundance vectors were CLR transformed and used for all downstream analysis. An RFC framework (Breiman, 2001) was then used to classify the samples by their diagnosis groups. The set of input features for the RFC consisted of the CLR-transformed sample relative abundance vectors, the metadata available in all cohorts (sex, age), and the unique subject ID (used to account for replicates). For the RFC analysis, the Healthy-1 and Healthy-2 cohorts were grouped under one label (Healthy) to create a single healthy control group to compare to the IBD and non-IBD sample groups thus allowing us to identify important features that distinguish between diagnosis groups rather than cohort in a more robust manner (Pasolli et al., 2016; Thomas et al., 2019). The RFC was then trained on the taxonomic profiles as well as the metadata available for all cohorts. While RFC’s provide feature importance’s based on the features’ contribution to classification of the given label, there is no statistical significance attached to these importance’s. To assess statistical significance of the features a random noise column was generated and added to the data (see section “Materials and Methods”). The species that were ranked as significantly more important than the random noise column were designated as the ‘signature species’ and used for all downstream analyses. A Mann–Whitney *U* test and Benjamini–Hochberg (BH) multi-test correction was used to compare the differential abundance of the signature species within IBD to all other groups individually.

Bacterial species that were significantly differentially abundant in IBD, relative to every other sample group, were designated as differentially abundant. Next, a GGM framework (see section “Materials and Methods”) was used to construct the bacterial association networks from the relative abundance information of each sample group. Finally, the genomic functional capacity within each sample group was determined by using the TIGRFAM protein family database. The TIGRFAM counts for each signature species were weighted by the relative abundance of the species within each sample group and then CLR-transformed. A Mann–Whitney *U* test and BH multi-test correction was then used to compare the differential abundance of the TIGRFAM functions within IBD to the other groups to determine differences in functional capacity.

### Alpha-Diversity Analysis

**FIGURE 1:**
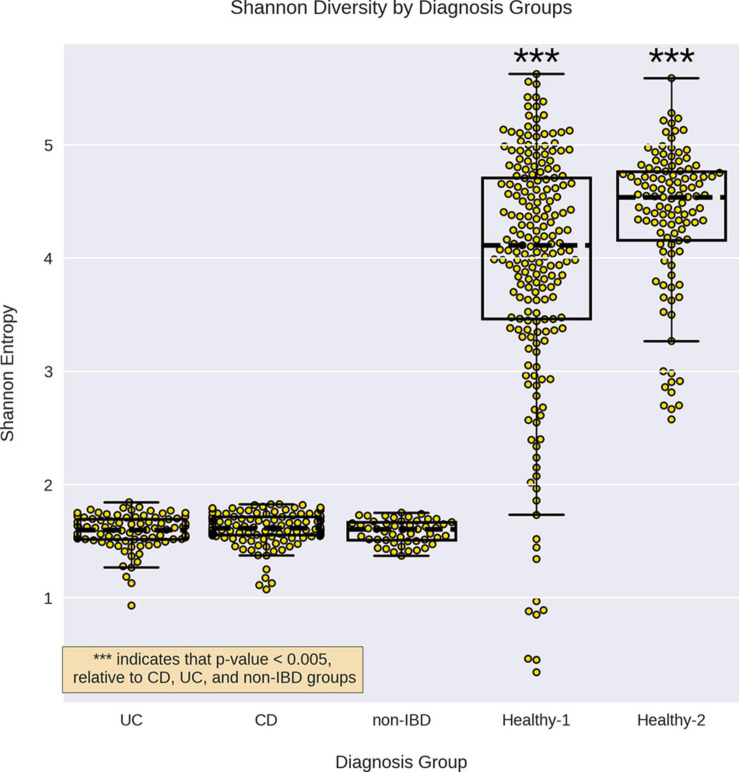
Alpha diversity for the different sample groups. Alpha diversity for each sample group by was calculated using Shannon entropy. The alpha-diversity for CD, UC, and non-IBD were not significantly different from each other but all three were significantly lower than the healthy cohorts. *** indicates a *p*-value < 0.0005 compared to CD, UC, and non-IBD, using a Mann–Whitney *U* test.

The non-IBD group displayed a similar alpha-diversity to the UC and CD groups, however, the external healthy cohorts displayed significantly higher alpha-diversities than all other groups (Figure 1). When examining the effect of cohort read-depth on alpha-diversity, we did not observe any significant correlation between read-depth and alpha-diversity (Supplementary Material 1). Notably, the Healthy-2 cohort displayed lower read-depth on average, relative to the IBDMDB cohort, but displayed significantly higher alpha-diversity.

### Intrapersonal Dissimilarity

**FIGURE 2:**
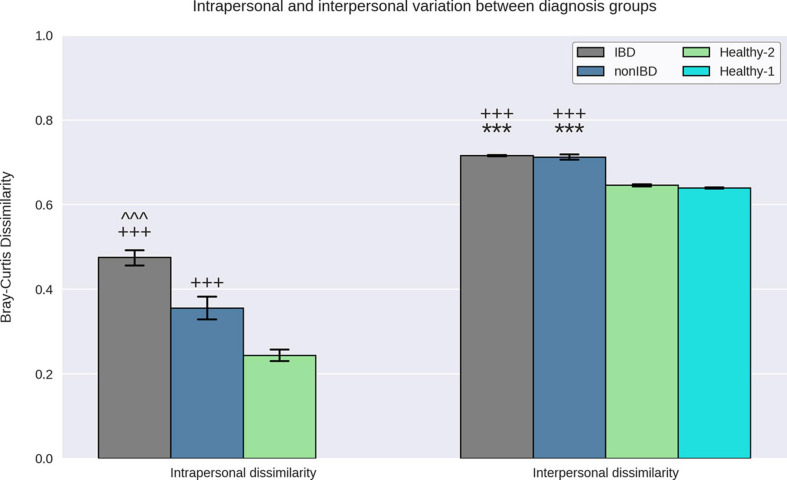
Interpersonal and intrapersonal variation. Bray–Curtis dissimilarities between replicate samples from the same subject were used to quantify intrapersonal variation while the Bray–Curtis dissimilarities between samples from different subjects were used to quantify interpersonal variation. IBD samples demonstrated elevated intrapersonal variation relative to non-IBD and Healthy-2 samples and non-IBD samples demonstrate elevated intrapersonal variation relative to Healthy-2 samples. IBD and non-IBD samples both demonstrated elevated interpersonal variation relative to the Healthy-1 and Healthy-2 samples. It was observed that replicate samples from the same subject were significantly more similar to each other than to samples from other subjects indicating that all diagnosis groups exhibited greater interpersonal variation than intrapersonal variation. *** Indicates *p*-value < 0.0005, relative to Healthy-1. +++ indicates *p*-value < 0.0005, relative to Healthy-2. ^^^ indicates a *p*-value < 0.0005, relative to non-IBD.

When examining intrapersonal dissimilarity, it was noted that samples from the same subject were significantly more similar to each other than they were to samples from other subjects (Figure 2). This trend was constant for every diagnosis group that could be tested (Healthy-1 cohort did not utilize replicates) and was statistically significant every time. Furthermore, it was observed that IBD samples demonstrated the highest levels of intrapersonal dissimilarity and were significantly higher than both non-IBD samples and Healthy-2 samples. Interestingly, the intrapersonal dissimilarity of non-IBD samples fell between the IBD and the Healthy-2 samples.

### Interpersonal Dissimilarity

To quantify how different the gut microbiota of samples within a specific diagnosis group are, we examined the interpersonal dissimilarity. Once again, the IBD samples exhibited the highest levels of dissimilarity when examining the interpersonal dissimilarity (Figure 2). IBD sample interpersonal dissimilarities were significantly higher than the Healthy-1 and Healthy-2 samples but were not significantly different than the non-IBD samples. It was also noted that the non-IBD samples displayed significantly higher interpersonal dissimilarity, relative to the Healthy-1 and Healthy-2 cohorts.

### Taxonomic Analysis

**FIGURE 3:**
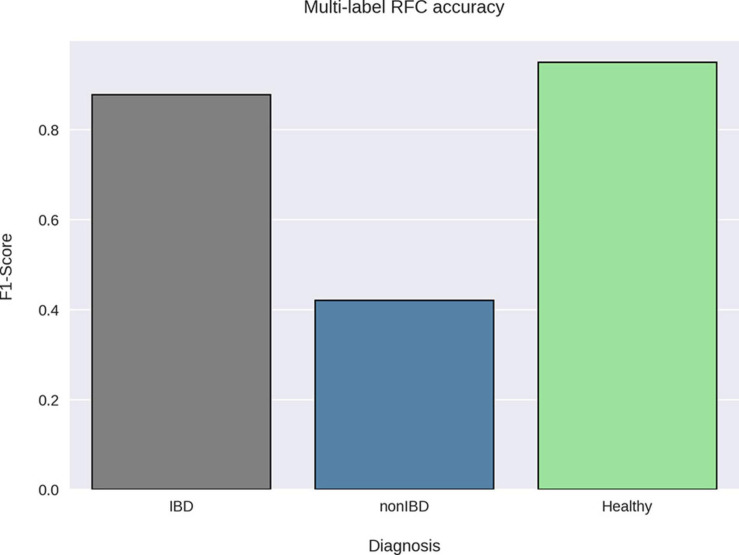
RFC classification accuracy by sample group. A random forest classifier was trained on taxonomic profiles and sample metadata using the diagnoses as the sample labels. The Healthy-1 and Healthy-2 cohorts were grouped together under one label (Healthy) as our goal was to classify samples by diagnosis. The F1-score was then calculated for each label using the precision and recall of the best-performing RFC model via threefold cross-validation. The RFC was able to accurately classify healthy and IBD samples using their taxonomic profiles but was not able to accurately classify non-IBD samples.

**FIGURE 4:**
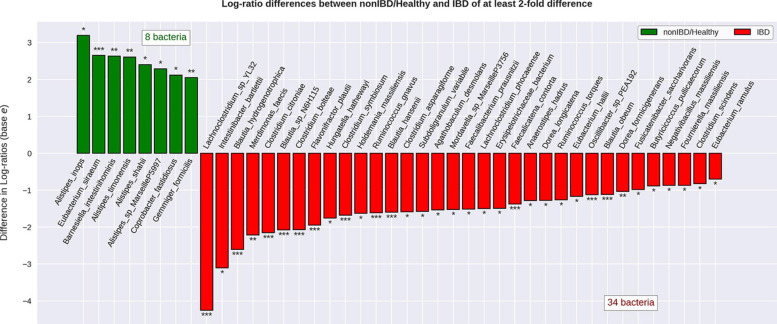
Differential abundance of bacterial species when comparing IBD to non-IBD and healthy groups. Relative abundance values were CLR-transformed and differential abundance was calculated for IBD compared to non-IBD, IBD compared to Healthy-1, and IBD compared to Healthy-2. The species that were significantly differentially abundant in IBD relative to every single other group were considered to be significantly differentially abundant resulting in 42 significantly differentially abundant bacterial species. Of these 42 species, 34 were found to be significantly more abundant in IBD relative to every other group and 8 bacterial species were found to be significantly less abundant in IBD, relative to every other group. The transformed relative abundances were then averaged and displayed under one label (non-IBD/Healthy) for ease of visualization. * Indicates a *q*-value < 0.05. ** Indicates a *q*-value < 0.01. *** Indicates a *q*-value < 0.001.

When attempting to classify all different diagnoses (CD, UC, non-IBD, and healthy) using the RFC, it was noted that CD and UC samples were often misclassified as one another (CD as UC or *vice versa*) which contributed to the modest RFC classification accuracy (weighted average F1-score: 0.79) (Supplementary Material 2a). After combining the CD and UC diagnoses into the IBD sample group, the RFC was able to distinguish between the various cohorts with higher average accuracy (weighted average F1-score: 0.87) (Figure 3). Notably, the non-IBD group was difficult to distinguish, and these misclassifications were split between IBD and healthy controls implying that the non-IBD group had a heterogeneous composition in which some samples resembled healthy samples and others resembled IBD samples (Supplementary Material 2b). The RFC model identified 122 important features with the ‘age’ feature demonstrating the greatest feature importance. The ‘unique subject ID’ feature was also an important feature but was ranked 99/122 according to feature importance. The remaining 120 important features were bacterial species. The CLR-transformed relative abundances of these 120 species were then compared between IBD and non-IBD (internal control) resulting in 55 significantly differentially abundance species. Out of these 55 species, 42 were significantly differentially abundant in IBD relative to all three control groups (non-IBD, Healthy-1 Healthy-2) with a *q*-value < 0.05 and greater than a twofold difference (Figure 4). Of those 42 species, 34 were elevated in IBD and 8 species were elevated in the internal and external controls. All 42 of the above species were also found to be differentially abundant when utilizing the union of the 90% prevalent species for the differential abundance analysis.

Out of the 34 species elevated in IBD, only the *Clostridium* (five species) and *Blautia* (four species) genera displayed more than two species elevated (Supplementary Material 4).

### Bacterial Association Networks

**FIGURE 5:**
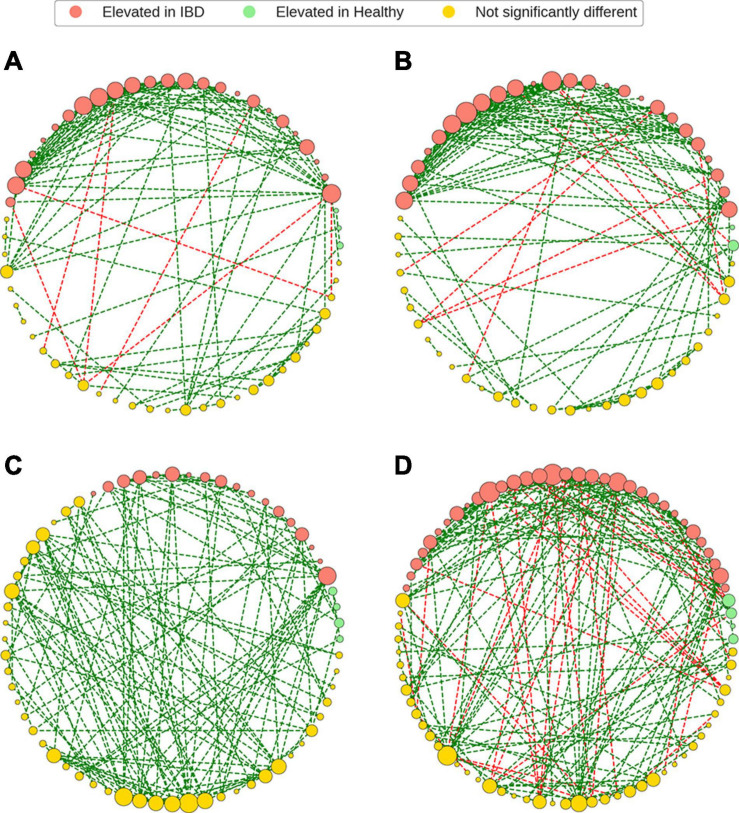
Gut microbiome bacterial species association networks. The GGM framework was used to generate bacterial association networks from CLR-transformed relative abundances. The bacterial species (nodes) were colored based on the differential abundance analysis and the node sizes were based degree of each node within the network. Bacterial species that were elevated in IBD were still present, and in high degree, in the non-IBD and healthy networks. It was also noted that the most common constituents of the bacterial association networks were bacteria that were not significantly differentially abundant in IBD, relative to the healthy and non-IBD groups. Finally, the IBD networks demonstrated more negative edges when compared to the non-IBD and healthy groups. **(A)** IBD network, **(B)** non-IBD network, **(C)** Healthy-1 network, **(D)** Healthy-2 network.

Bacterial species elevated in IBD had non-zero degree in all bacterial association networks (Figure 5). While these nodes were elevated in IBD, they still maintained a higher-than-average number of associations within all networks (Supplementary Material 5). It was observed that while the nodes elevated in IBD display higher than average degree, most nodes within each network were composed of species that were not significantly different between IBD and the control groups (IBD: 52.5%, non-IBD: 52.6%, Healthy-1: 65.6%, Healthy-2: 53.7%) (Supplementary Material 6). When examining the most important species within the network, defined as the species with the ten highest Eigenvector centralities, a measure of relative importance or influence of nodes, within a network, all but two of the ten species were found in the top-10 important species non-IBD or healthy networks (Supplementary Material 7) (Newman, 2006). While there was a large amount of overlap, there were also 56 associations that are unique to the IBD network (Supplementary Material 8). The vast majority of these associations (85.71%) involved species that were elevated in IBD.

### Differences in Functional Capacity

**FIGURE 6:**
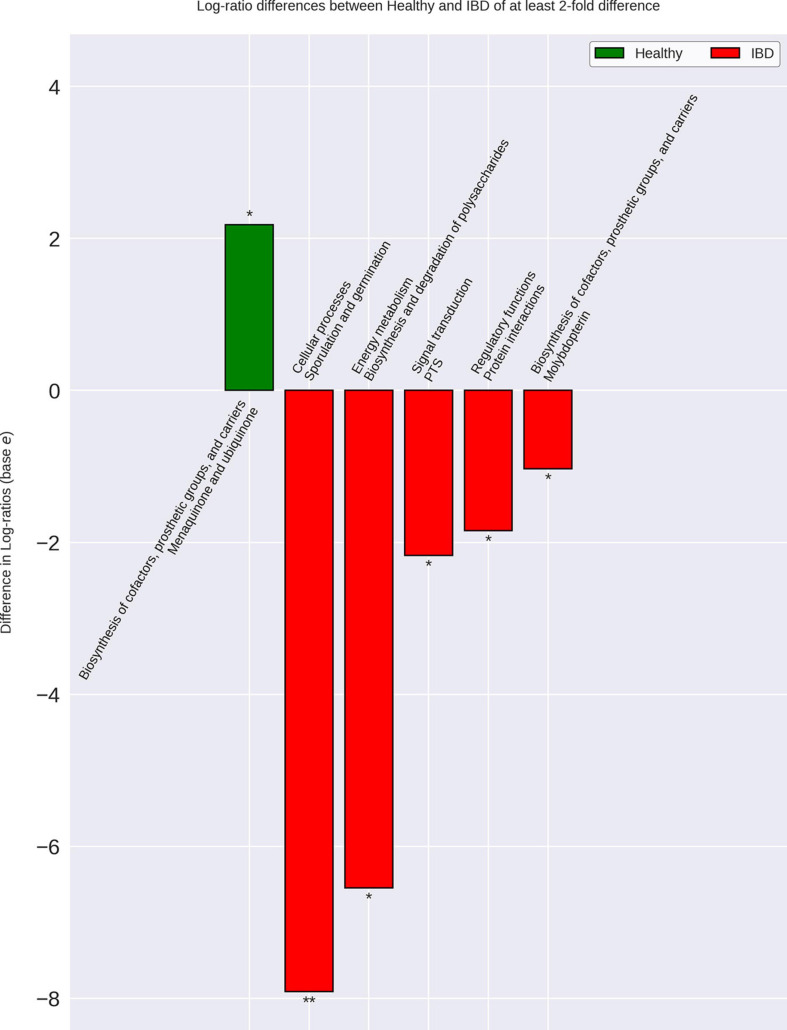
Differences in IBD gut microbiome functional capacity. Genomic functional capacity was determined by using the TIGRFAM protein family database. The counts for each TIGRFAM within a bacterial species were weighted by the relative abundance of the bacterial species within each group. The CLR-transformed relative abundance of the TIGRFAM’s within IBD were then compared to the non-IBD, Healthy-1 cohort, and Healthy-2 cohort individually. The differentially abundant TIGRFAM’s were then summed based on their roles, according to the TIGRFAM database. There were no differences between the IBD and non-IBD gut microbiome functional capacities. There were six significantly differentially abundant protein family roles when comparing IBD to the Healthy-1 cohort that were also found in the Healthy-2 cohort. These differences are implicated in important processes that may contribute to IBD-related symptoms such as diarrhea, intestinal bleeding, and increased intestinal permeability. The relative abundances of the Healthy-1 and Healthy-2 cohort TIGRFAM roles were averaged for ease of visualization. * Indicates a *p*-value < 0.05. ** Indicates a *p*-value < 0.01.

Analysis of the genomic functional capacities of the different cohorts demonstrated six significant differences with greater than twofold fold change between the IBD cohort and all other cohorts (Figure 6). IBD samples displayed elevated relative abundance of protein families involved in sporulation and germination, synthesis and degradation of polysaccharides, signal transduction, regulatory protein interactions, and molybdopterin biosynthesis. The IBD samples also displayed reduced relative abundance of protein families involved in menaquinone and ubiquinone synthesis. Out of the 34 bacterial species elevated in IBD, 13 were previously found to be associated with IBD, CRC, IBS, obesity, or rectal bleeding and 8 of the 13 species were found to have multiple roles (Supplementary Material 9). A particular interest within this group of 13 bacteria were the species that have been studied *in vitro* or *in vivo* and found to potentially play a role in IBD such as *Ruminococcus gnavus, Flavonifractor plautii, Clostridium symbiosum*, and *Clostridium scindens.* Out of the 21 remaining species, 16 were novel potential markers for IBD, 1 was previously found to be reduced in UC, and 4 were previously found to be elevated in healthy samples.

## Discussion

This study identified numerous differences in taxonomic profiles, bacterial association networks, and genomic functional capacity between the IBD gut microbiome and the control gut microbiomes. Furthermore, our findings were corroborated by multiple external cohorts, and were generated using techniques and analyses that account for the compositionality of sequencing data. To our knowledge, this is the first study to utilize multiple external cohorts from a similar geographic region to corroborate comparisons between the internal control group and the diseased group in an analysis of the gut microbiome while also utilizing a compositionally robust methodology. Additionally, we demonstrated that bacterial species whose relative abundance is elevated in IBD are also present in the healthy microbiomes and maintain an important position in the healthy and IBD bacterial association networks implying that these species play an important role in the gut microbiome. However, these elevated bacteria are also often implicated in mucin degradation, immune system modulation, antibiotic resistance, and modulation of inflammation and their over-abundance may dysregulate these important processes possibly contributing to IBD pathogenesis and IBD-related symptoms.

We found that the IBD samples had alpha-diversities similar to internal controls (non-IBD), but significantly lower than external healthy controls. While it has previously been noted that IBD samples have lower alpha-diversity than healthy controls, we believe this may be due to the convenience selection of internal controls (Frank et al., 2011; Gevers et al., 2014; Sheehan et al., 2015). As reported in Lloyd-Price et al. (2019) the internal controls (non-IBD) consisted of “patients [who] were approached for potential recruitment upon presentation for routine age-related colorectal cancer screening, work up of other gastrointestinal (GI) symptoms, or suspected IBD, either with positive imaging (for example, colonic wall thickening or ileal inflammation) or symptoms of chronic diarrhea or rectal bleeding” However, due to ∼75% of internal control samples being derived from subjects below the age of 45 (the earliest recommended age for colorectal cancer screening without personal or family history of colon cancer), it is presumed that the majority of these subjects presented with GI distress (Lloyd-Price et al., 2019; Supplementary Material 10).

When examining the replicates present in the IBDMDB and Healthy-2 cohorts, it was noted that subjects diagnosed with IBD demonstrated increased temporal variability, as measured by the intrapersonal dissimilarity, when compared to non-IBD samples and Healthy-2 samples. This has been previously demonstrated when comparing CD and UC to non-IBD controls and has been posited to be caused by the inflammation and decreased intestinal transit time experienced by IBD patients as well as the medications and lifestyle changes employed to manage IBD (Clooney et al., 2021). It was also noted that the IBD and non-IBD samples displayed greater subject-to-subject variability relative to Healthy-1 and Healthy-2 samples. The relatively elevated temporal stability and subject-to-subject variability indicates that the gut microbiota of our IBD samples displayed increased heterogeneity, relative to healthy controls. This has also been previously demonstrated in pediatric IBD patients and is believed to be caused by a depletion of core microbes, possibly due to inflammation and IBD therapies (Schirmer et al., 2018).

Much like the original publication utilizing the IBDMDB cohort (Lloyd-Price et al., 2019), differentiating between the taxonomic profiles of IBD from non-IBD samples was difficult. In our study, using the RFC to classify IBD and non-IBD samples yielded many misclassifications in which non-IBD samples were consistently classified as IBD. The non-IBD samples were also misclassified as healthy. This split of RFC misclassifications for non-IBD samples indicates that the non-IBD group consists of a heterogeneous group that resembles both the IBD group, such as the subjects presenting with GI distress, and the healthy groups, such as the subjects presenting for routine screenings. It was also noted that the RFC utilizing the taxonomic profiles misclassified CD samples as UC samples and *vice versa*. This has also been previously demonstrated in other studies utilizing shotgun sequence data and is indicative of the high similarity demonstrated between the taxonomic profiles of the CD and UC gut microbiomes (Moustafa et al., 2018; Franzosa et al., 2019). This difficulty of distinguishing between the CD and UC taxonomic profiles is possibly due to similar biological processes involved in both diseases, especially when comparing the inflammatory processes underlying both CD and UC (Olsen et al., 2007).

The RFC was able to distinguish between the external healthy cohorts and the IBD samples consistently and accurately, most likely due to these cohorts being composed of samples with no reported or overt disease. Our modified RFC framework also allowed us to distinguish bacterial species that had a higher ranking than the random feature, based on the RFC feature importance’s. These species were then used for differential abundance analysis, and network construction. While there was difficulty distinguishing the non-IBD sample taxonomic profiles from the IBD and healthy sample taxonomic profiles utilizing the RFC, we were able to distinguish 55 bacterial species that were significantly differentially abundant between the IBD and non-IBD groups. Of these 55 species, 42 were differentially abundant with a greater than twofold change in the external cohorts as well.

The bacterial association networks revealed that while some bacteria were found to be elevated in IBD, they were still present in non-zero degree in non-IBD and healthy networks. As a matter of fact, the species elevated in IBD displayed higher than average degree in all networks except for the Healthy-1 network. Furthermore, when examining the most important nodes (top-10 eigenvector centrality) within the IBD network, 8 out of the 10 species were also found in the top-10 eigenvector centrality (EVC) nodes of the healthy networks but all 10 of the top EVC species were found to have relative abundances that are elevated in IBD samples. The presence and importance of species that are elevated in IBD appears to be ubiquitous throughout all networks implying that while these species have an increased relative abundance in IBD, they still play integral roles within the non-IBD and healthy microbiomes, and that it is their over-abundance and not mere presence that plays an important role in IBD. Interestingly, while bacteria with elevated relative abundances in IBD were present and appeared to play an important role in the non-IBD and healthy networks, they also demonstrated many associations unique to the IBD network illustrating that some bacterial species can associate with different bacteria due to factors other than just the presence of the bacteria. This implies that other factors, such as host genetics, host diet, intestinal environment, or medications may lead to the unique associations (Pérez-Gutiérrez et al., 2013; Ohland and Jobin, 2015).

It was also noted that most species within each network were not differentially abundant between IBD and the control groups (IBD: 52.5%, non-IBD: 52.6%, Healthy-1: 65.6%, Healthy-2: 53.8%). This is an interesting finding demonstrating that most gut microbiome network constituents are similar in relative abundance between healthy and IBD gut microbiomes. Furthermore, we observed that these non-differentially abundant bacteria accounted for greater than 60% of the relative abundances in all groups (IBD: 62.6%, non-IBD: 70.5%, Healthy-1: 74.6%, Healthy-2: 64%). Most bacterial association networks and most of the gut microbiome were composed of bacteria that are not significantly differentially abundant between the IBD and control gut microbiota indicating that the differences in the IBD gut microbiota are not wide-spread and appear to be limited to a set of bacterial species with significantly higher relative abundance. Interestingly, it was also observed that the majority of negative associations found in all networks were associated with species displaying elevated relative abundance in IBD samples (IBD network: 100%, non-IBD: 100%, Healthy-1: no negative edges, Healthy-2: 81.6%). This finding indicates that the bacterial species elevated in IBD may play an important role in maintaining stability, possibly by preventing positive feedback loops, but due to their overabundance in IBD they may contribute to reducing the diversity of the gut microbiome in IBD samples (Coyte et al., 2015).

When analyzing the protein family relative abundances in each cohort, we were not able to identify any statistically significant differences in functional roles between the IBD and non-IBD group. However, we were able to find six significantly different functional roles between the IBD group and each of the external control cohorts. Notably, the protein family role most elevated in IBD, relative to external healthy controls, was associated with functions related to sporulation and germination. While sporulation in the context of GI disease is most often associated with *Clostridium difficile*, many members, especially pathogens, of the *Clostridia* genus have been found to utilize sporulation which is in-line with our data demonstrating that the *Clostridium* genus is the most commonly elevated genus in IBD (Hookman and Barkin, 2009; Shen et al., 2019). Our analysis also demonstrated that protein families involved in polysaccharide metabolism were elevated in IBD. This may be due to the increase in relative abundance of some bacteria that inhabit the intestinal mucosa and degrade mucin to derive glycans as an energy source, such as *Ruminococcus gnavus* and *Clostridium symbiosum* (Bernalier-Donadille, 2010; Desai et al., 2016; Hall et al., 2017). It was also found that protein families involved in molybdopterin synthesis were significantly elevated in IBD. Molybdopterin is an important co-factor for nitrate reductase, which reduces nitrate to nitrite (Moreno-Vivián et al., 1999). Previous research has identified nitrite as an important molecule in the regulation of mucosal blood flow, intestinal motility, and mucus membrane thickness, however, it believed that an over-abundance of nitrite can have deleterious effects on commensal bacteria and has been shown to be associated with IBD as well as with increased bleeding (Lidder and Webb, 2013; Park et al., 2013; Tiso and Schechter, 2015). This may indicate that an increase in nitrate reduction (leading to increased nitric oxide levels) can contribute to negative selection against commensal bacteria as well as contribute to increased propensity of intestinal bleeding in IBD. Nitric oxide, the main metabolite of nitrite, is also believed to be able to increase intestinal motility and lead to diarrhea (Kukuruzovic et al., 2003).

We also observed that protein families involved in the synthesis of quinones (menaquinone and ubiquinone) were reduced in IBD. Quinones are believed to be important growth factors for gut microbiota, especially for bacteria seen as commensals (Fenn et al., 2017). Humans are also unable to synthesize menaquinone (Vitamin K) and thus must ingest it or have it produced by commensal bacteria indicating that a reduction in vitamin K synthesis by the gut microbiota may lead to a reduction of vitamin K levels in IBD (Walther et al., 2013). In fact, IBD research has long noted that IBD patients present with lower vitamin K levels (Krasinski et al., 1985; Schoon et al., 2001). Due to the important role of vitamin K in blood clotting and calcium binding, this reduction on vitamin K has been used to explain common co-occurrences and symptoms of IBD such as osteoporosis and bleeding (Schoon et al., 2001; Agnello et al., 2014). Quinone synthesis appears to play an important role in maintaining host health and its reduction may contribute to the increased intestinal and rectal bleeding common in IBD.

Finally, we were able to identify specific bacterial species that are elevated in IBD and play important roles in fomenting inflammation, degrading mucin, and antibiotic resistance. *R. gnavus* and *C. symbiosum* are mucin-degrading bacteria that are found in healthy gut microbiomes but are shown to be elevated in IBD gut microbiomes (Crost et al., 2016). These bacteria may play an important role in preventing the over-secretion of mucus in healthy gut microbiomes, but their over-abundance may cause the mucus layers in the intestine to become too thin. We also identified *Flavonifractor plautii* as a species that was elevated in IBD. *F. plautii* has been found to degrade flavonoids, an important anti-inflammatory mediator in humans and mice (Musumeci et al., 2020). The over-abundance of *F. plautii* can lead to low levels of flavonoids which has been shown to lead to increased inflammation, particularly in the gut microbiome (Gupta et al., 2019). We also identified *Clostridium scindens* as a novel association with IBD. It was previously noted that *C. scindens* is associated with the generation of secondary bile acids (SBAs) in the gut microbiome (Marion et al., 2019). While SBAs play an important role in the healthy gut microbiome, an over-abundance of SBAs may lead to cell-membrane disruption, reactive oxygen species generation, cellular DNA damage, and colorectal cancer (Payne, 2008; Perez and Britz, 2009; Ajouz et al., 2014). *R. gnavus, C. symbiosum, F. plautii*, and *C. scindens* are key examples of bacterial species that are present, and potentially important, in healthy microbiomes but may exhibit deleterious effects on host health when they become over-abundant.

While we attempted to mitigate as many confounders under our control as possible, there are still limitations to be cognizant of within our study. One particularly important limitation stems from the relatively low number of subjects present in the datasets we utilized. We previously demonstrated that as the sample-to-taxa ratio increases, our network inference framework generates better predictions (Loftus et al., 2021), however, due to the low number of unique individuals it was necessary to construct the networks using the replicates as individual samples. While we have demonstrated that the intrapersonal variation is lower than the interpersonal variation, we do not believe that this has a negative effect on the accuracy of the networks inferred. In our analysis, we have assumed that all samples from a cohort are generated using the same underlying covariance structure; that is, for each cohort, there is a single multivariate Gaussian distribution associated with it, and this distribution has an unknown covariance matrix whose parameters we estimate using the GGM framework. Under this (simplistic) assumption, it is reasonable to include subject sample replicates for network inference. Another limitation is that our analysis focused on abundant species (present in > 90% of samples within a diagnosis group) to mitigate the high dimensionality present in gut microbiome analysis, however, some species with low-prevalence may still play important roles in the gut microbiome (Zhang et al., 2016). Also, there appeared to be a bias toward samples from younger subjects in the IBDMDB cohort. Approximately half of (46.6%) IBDMDB samples were derived from subjects below the age of 18 (Supplementary Material 10a) and the youngest subject was 6 years of age. In contrast, no subjects in the Healthy-2 cohort were below the age of 18 (Supplementary Material 11). While we did not have access to the metadata (other than sex) of the Healthy-1 cohort, it was previously published that all subjects fell between the ages of 18–40 (Methé et al., 2012). The feature ‘age’ also displayed the greatest feature importance during classification according to our RFC framework, indicating that there was a non-trivial difference in the ages between the diagnosis groups. It has been previously observed that the taxonomic profiles of individuals begin to resemble adult configurations by 3 years of age, indicating that the bias is unlikely to contribute to major differences in the taxonomic profiles and may just be indicative of the younger age of subjects in the IBDMDB study (Yatsunenko et al., 2012). However, the same study did note that while interpersonal variation greatly decreased after 3 years of age, it was still significantly higher in subjects between the ages of 3–17, relative to adults (18+ years of age), which may explain some of the difference in interpersonal variability observed between IBD and non-IBD samples, relative to the Healthy-1 and Healthy-2 samples. Finally, it was noted that there was a greater proportion of female subjects in the Healthy-2 cohort relative to the Healthy-1 and IBDMDB cohorts (Supplementary Material 12). This does not appear to impact the classification results, however, as the RFC did not find the features ‘sex’ to be more important than random noise.

By utilizing two external control cohorts, we were able to identify and corroborate 34 bacterial species whose relative abundance is significantly elevated in IBD. These species appear to play important roles in all bacterial association networks (IBD, non-IBD, and external healthy controls) implying that while an elevation of their relative abundance is associated with IBD, they are also important to the function of healthy gut microbiomes. Furthermore, we identified important differences in functional capacities between IBD and the healthy controls that may contribute to the onset or exacerbation of IBD-related symptoms such as diarrhea, intestinal bleeding, mucin degradation, and intestinal inflammation. Finally, we were able to corroborate many of the bacterial species we identified as elevated in IBD using previously published research and identified 17 novel bacterial species that may play an important role in IBD. To the best of our knowledge, we are the first to corroborate our analysis of the IBD gut microbiome by using external cohorts from the same geographic region (US) allowing us to generalize our findings to the population rather than only our study groups. Furthermore, we were able to illustrate important potential mechanistic links between the bacterial species elevated in the IBD gut microbiome and IBD-related symptoms. Finally, we identified differences in the genomic functional capacity of the IBD microbiome that bridges previous findings in IBD and IBD-related symptoms with the gut microbiome.

## Data Availability Statement

Publicly available datasets were analyzed in this study. This data can be found here: https://github.com/syooseph/YoosephLab/blob/master/MicrobiomeNetworks/IBD/.

## Author Contributions

SH initiated the study, wrote the manuscript, and created the figures. ML and SY assisted in study design. SH conducted the taxonomic, network, and functional analysis with assistance from ML and SY. All the authors reviewed the manuscript.

## Conflict of Interest

The authors declare that the research was conducted in the absence of any commercial or financial relationships that could be construed as a potential conflict of interest.

## Footnotes

1 www.ncbi.nlm.nih.gov/assembly/GCF_000001405.38
