# Supplementary material for: Linking Inflammatory Bowel Disease Symptoms to Changes in the Gut Microbiome Structure and Function
Source: Front Microbiol. 2021 Jul 19;12:673632. doi: 10.3389/fmicb.2021.673632 (PMC8326577; doi:10.3389/fmicb.2021.673632)
Supplement: Supplementary file 1 [file Data_Sheet_1.zip › Data Sheet 1.PDF]

Supplementary Information

Supplemental 1: Effect of read-depth on alpha diversity.

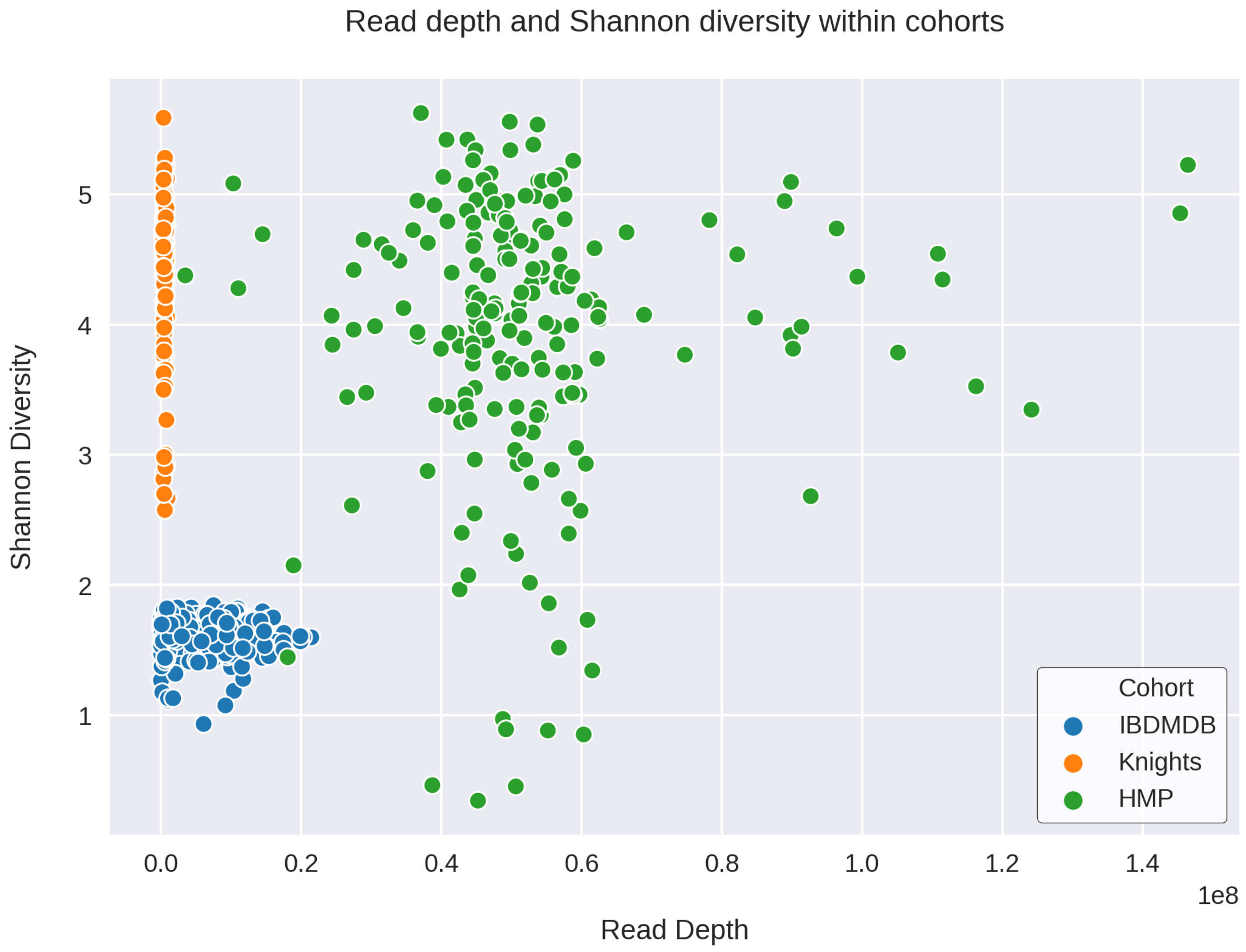

Caption: To illustrate that the alpha-diversity value differences were not due to read depth, the read depth was plotted against the alpha-diversity (Shannon entropy). Read depth did not appear to have an effect on alpha-diversity and notable the Healthy-2 cohort had lower read depth but displayed greater alpha-diversity.

Supplemental 2: Classification accuracy and misclassification before combining CD and UC into one IBD group.

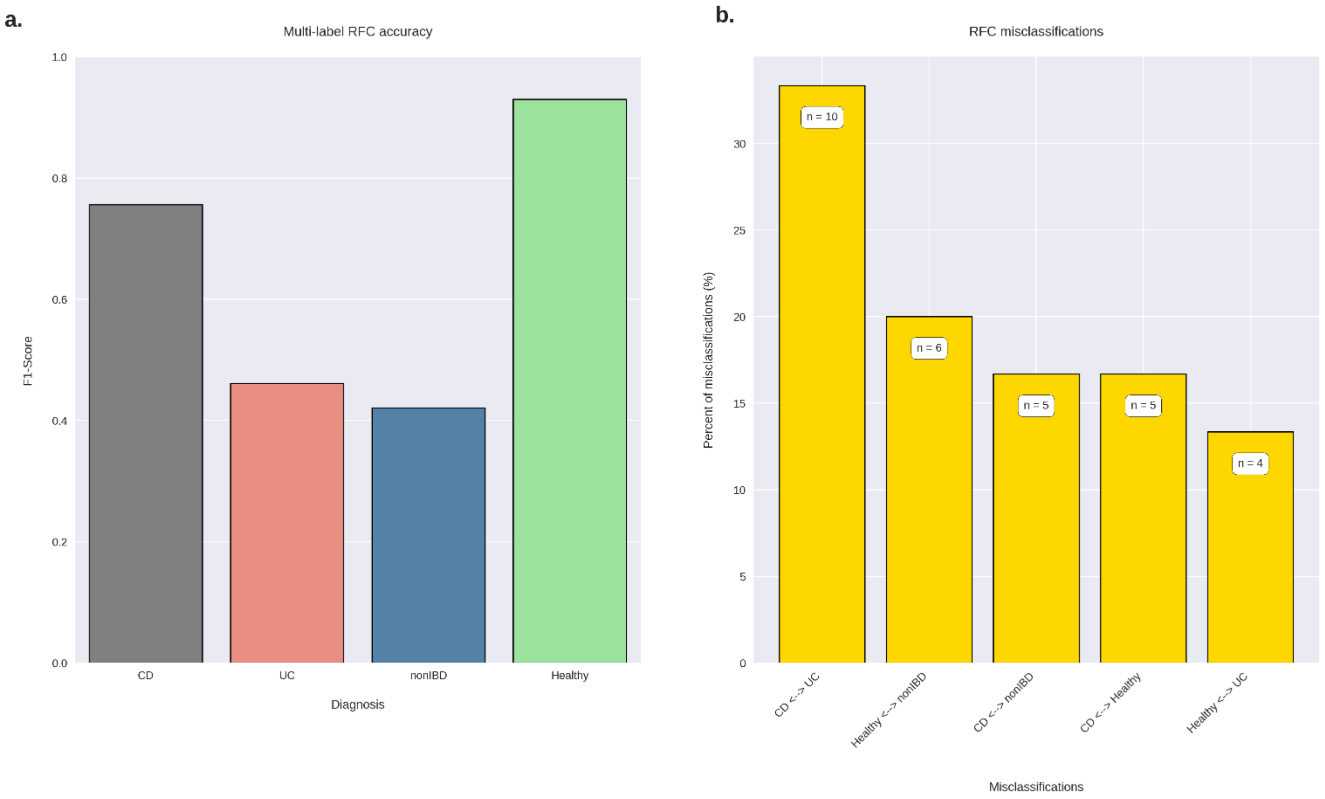

Caption: An RFC was trained on the taxonomic profiling data and metadata (age, sex, unique subject ID) for all groups without combining the CD and UC groups into one group. **a.** The RFC demonstrated poor classification accuracy when attempting to distinguish all groups (CD, UC, non-IBD, and Healthy), especially when attempting to classify the CD, UC, and non-IBD groups. **b.** The largest amount of misclassifications occurred due to the RFC classifying CD samples as UC and *vice versa* implying that the taxonomic profiles of both groups were very similar.

Supplemental 3: RFC misclassifications and accuracy after grouping CD and UC samples into one group (IBD).

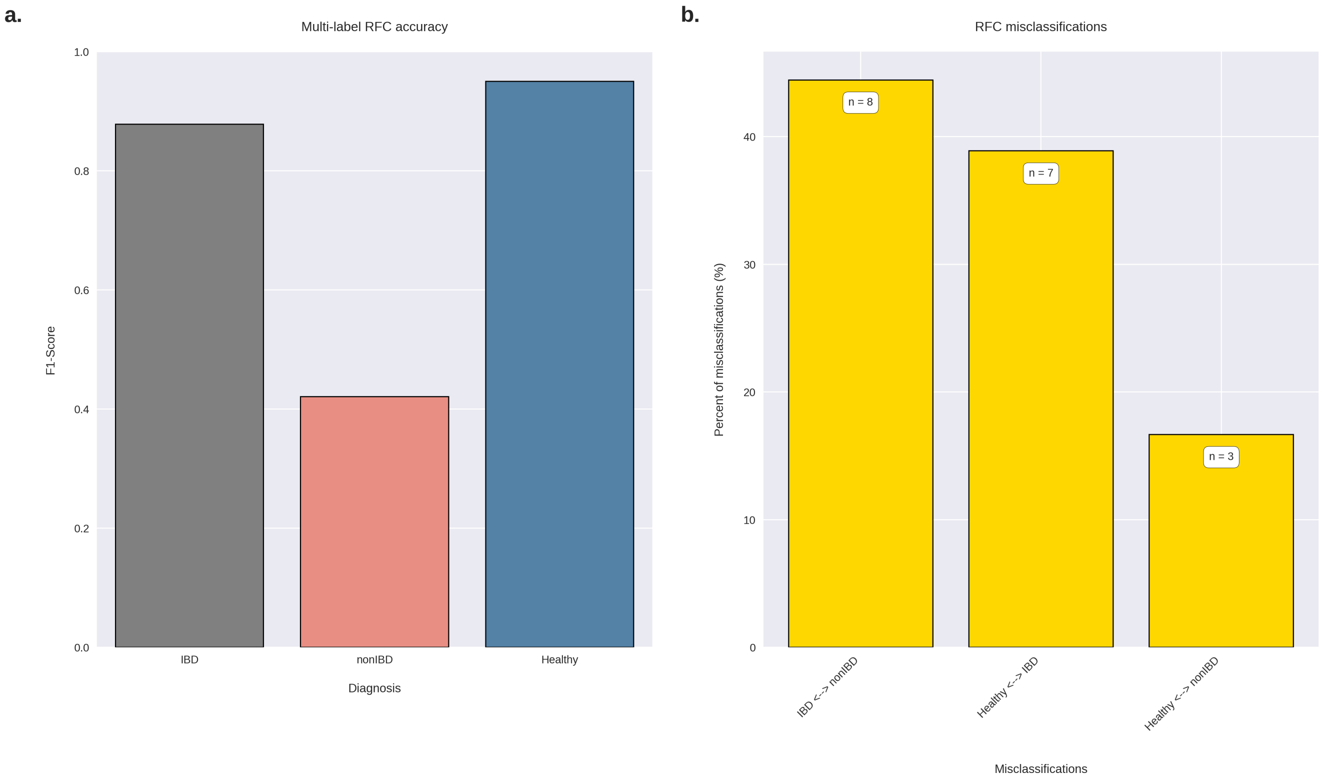

Caption: An RFC was trained on the taxonomic profiling data and metadata (age, sex, unique subject ID) for all groups after combining the CD and UC groups into one group, known as the IBD group. **a.** The RFC demonstrated better classification accuracy (weighted average of 0.87) compared to previous RFC without combining CD and UC samples under the IBD umbrella (weighted average of 0.79), however, non-IBD samples were still difficult to classify. **b.** The non-IBD samples were still consistently misclassified and were split (with a bias towards being classified as IBD) between being classified as IBD and Healthy samples.

Supplemental 4: Genera counts of bacterial species elevated in IBD samples.

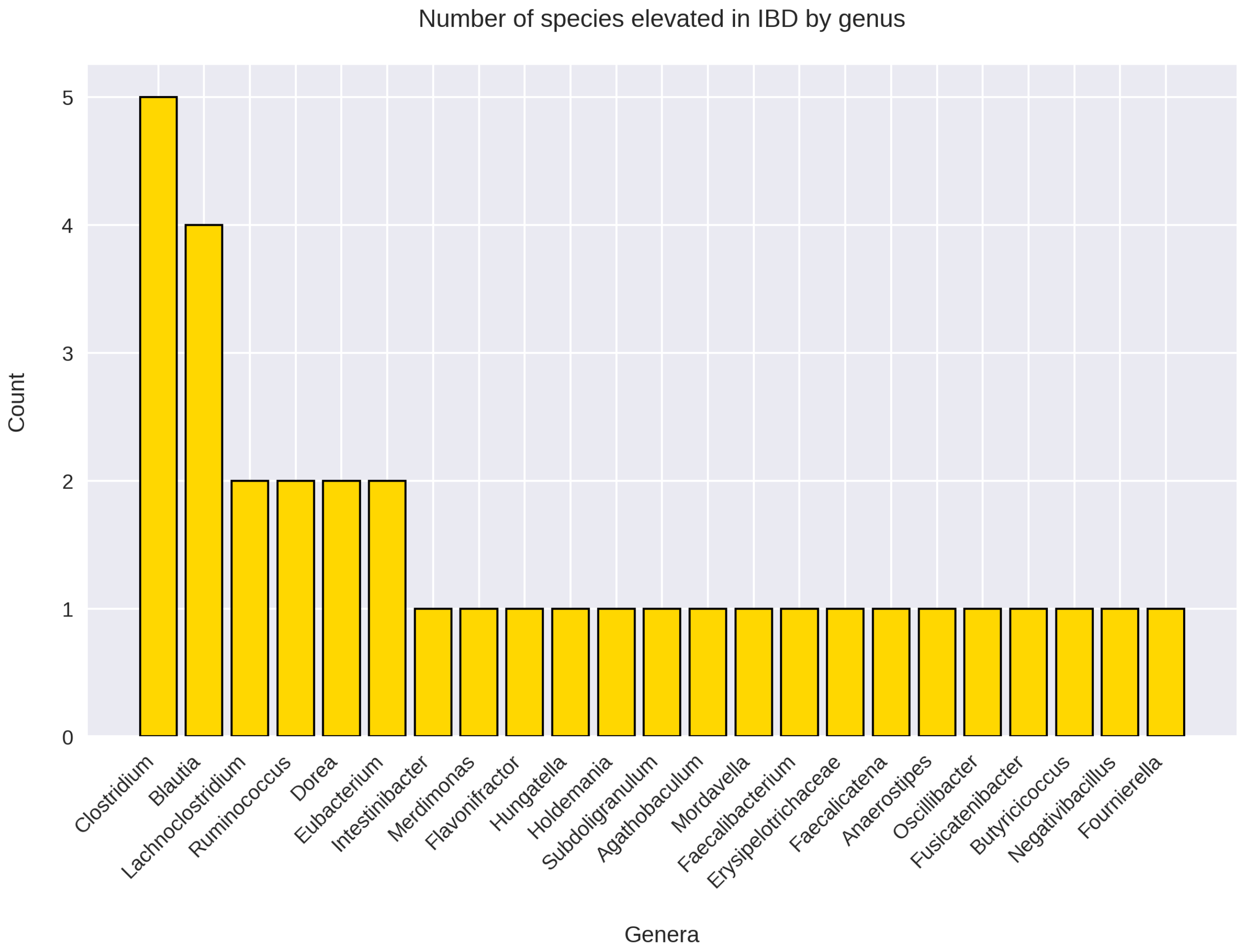

Caption: The genera of the bacteria that were elevated in IBD, according to the differential abundance analysis, were counted. *Clostridium* and *Blautia* were the most commonly elevated genera in IBD samples. *Lachnoclostridium, Ruminococcus, Dorea,* and *Eubacterium* were the only other genera to have more than one member elevated in IBD.

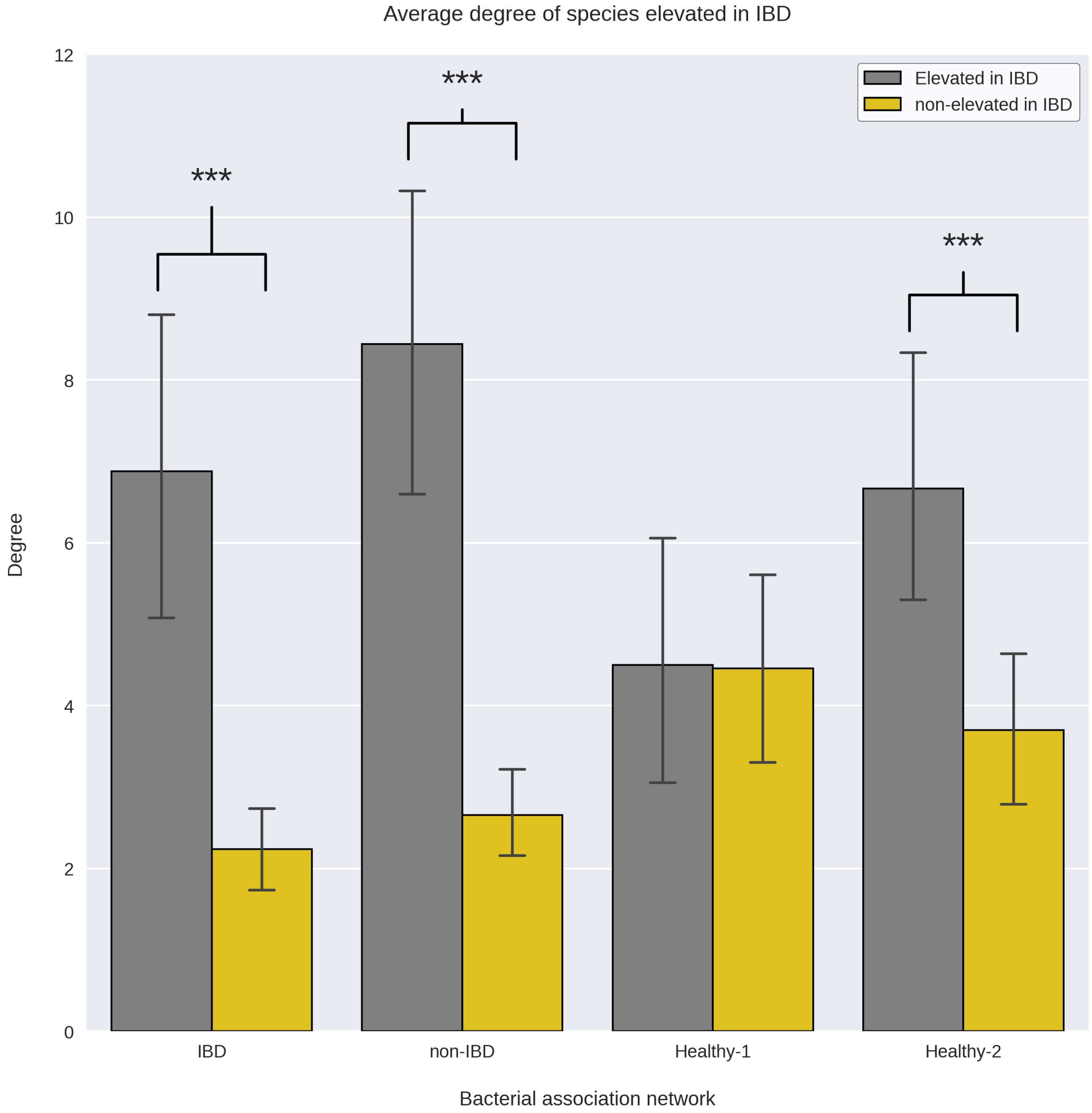

Supplemental 5: Average degree of bacterial species that are elevated in IBD within each network.

Caption: The average degree of the bacterial species that were elevated in IBD was calculated for each group and compared to the average degree of species not elevated in IBD (elevated in Healthy or not significantly different). On average, the species elevated in IBD displayed a higher number of connections (degree) within the bacterial association networks of all diagnosis groups and was significantly higher in 3 out of the 4 networks.

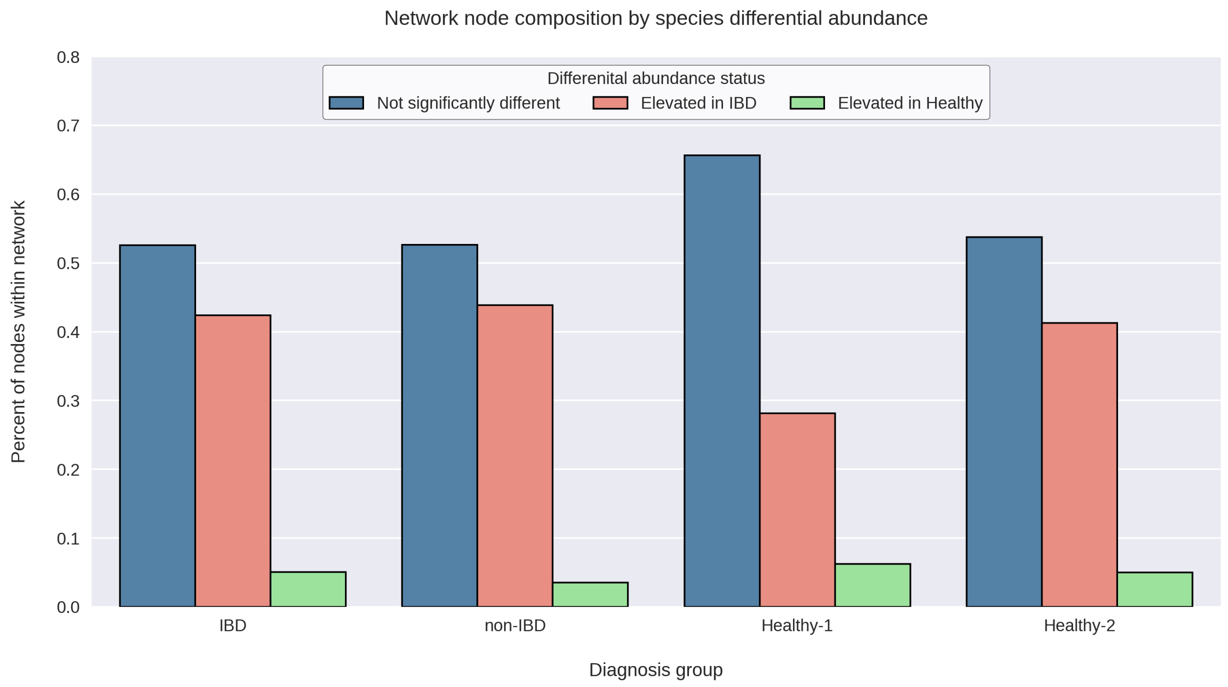

Supplemental 6: Network node compositions.

Caption: The Majority of nodes for each network are composed of species that are not differentially abundant between IBD and the other diagnosis groups.

Supplemental 7: Table of top-10 bacterial species in IBD network, according to Eigenvector centrality.

Caption: Eigenvector centrality (EVC) of each node (bacterial species) in the IBD network was calculated. The ten nodes with the highest EVC in IBD were also found in the top-ten EVC nodes of the non-IBD, Healthy-1, or Healthy-2 networks except for *Fusicatenibacter saccharivoran a*nd *Blautia hansenii.*

Supplemental 8: Unique associations within the IBD bacterial association network.

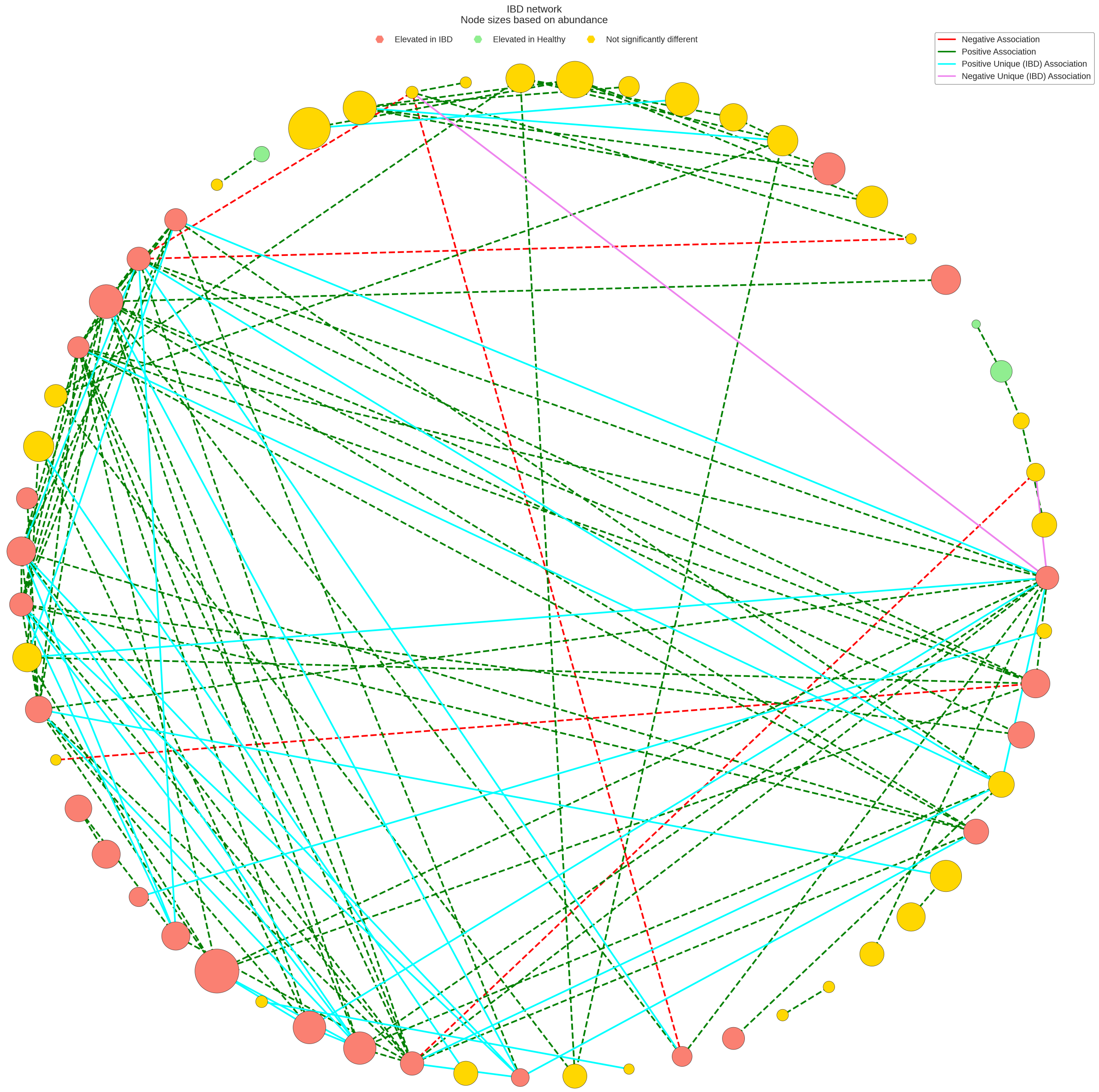

Caption: When comparing the structure of the IBD bacterial association network to all other networks, 56 associations were found that were unique to IBD networks only. The majority (85.7%) of these associations involved bacteria elevated in IBD. Even though the bacteria elevated in IBD are also present in the control networks, and in high degree, they do appear to demonstrate different associations in the IBD network.

Supplemental 9: Table of bacteria elevated in IBD (Included as additional file 1).

Caption: Bacterial species that were found to be elevated in IBD, according to the differential abundance analysis, underwent a literature search to determine if any previous research had been conducted on these bacterial species If research conducted on the bacterial species was found, the function, in the context of IBD, was stored, and the citations were listed. Keywords were created using the identified species function.

Supplemental 10: Age groups within the IBDMDB cohort and non-IBD samples.

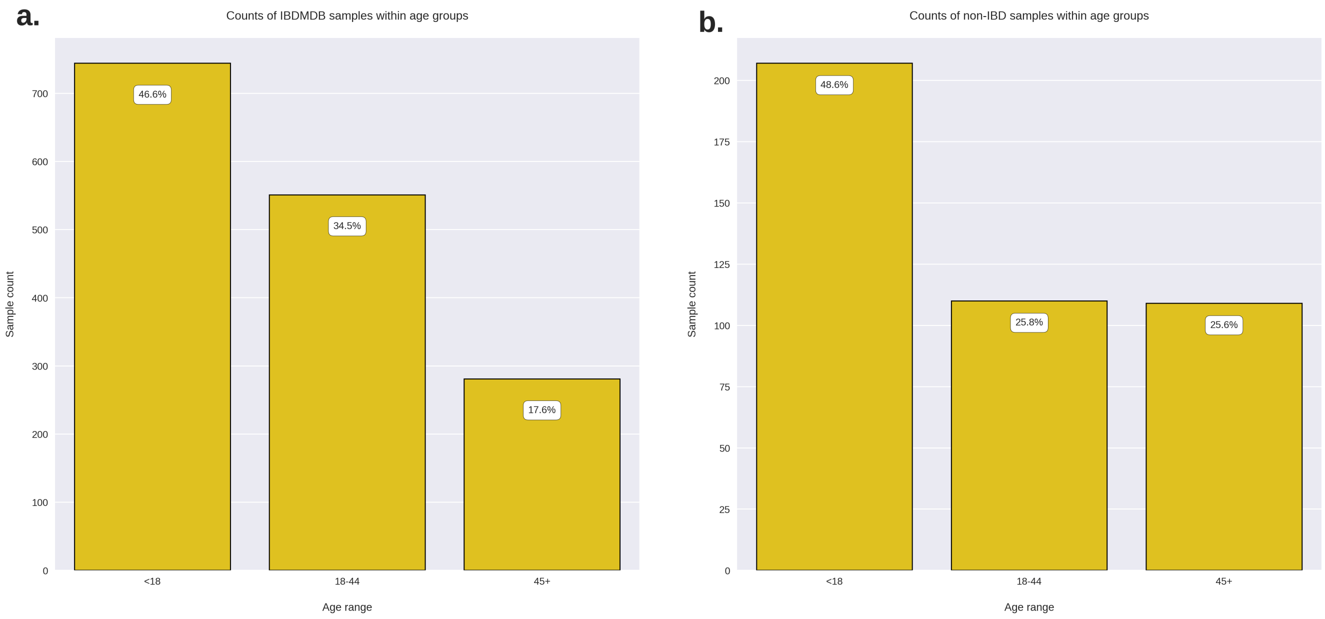

Caption: The counts of age ranges for subjects from the IBDMDB cohort were plotted. **a.** Almost half (46.6%) of samples in the IBDMDB cohort were from subjects below the age 18. **b.** The majority of non-IBD samples were derived from subjects that fell below the age of 45, the recommended age for colorectal cancer screening. Due to the description of subject recruitment from the original publication (Lloyd-Price et. al. 2019), it is presumed that the majority of the control group were comprised of samples that presented for GI distress or suspected IBD.

Supplemental 11: Age ranges of Healthy-2 samples.

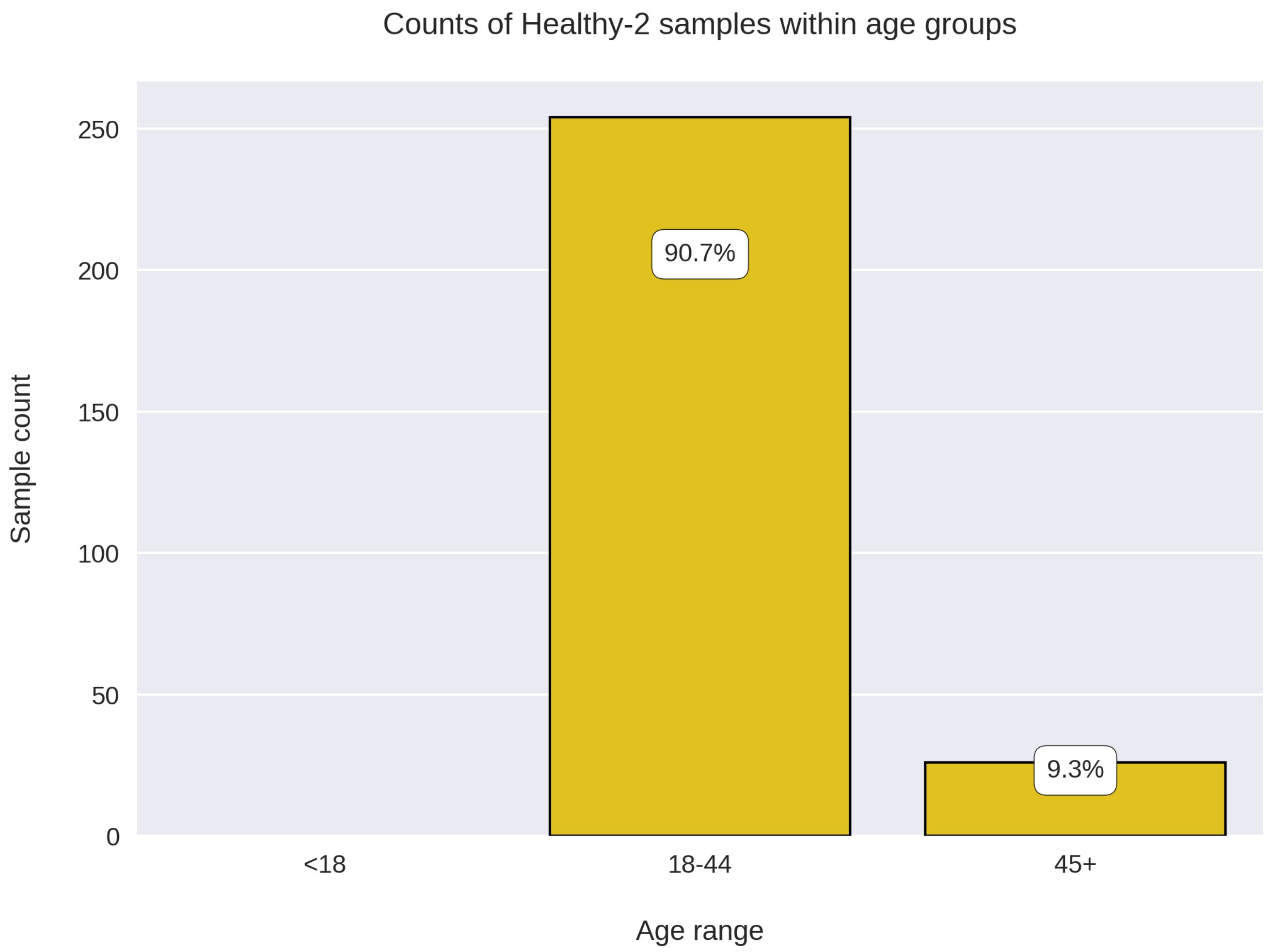

Caption: The age range for subjects of the Healthy-2 cohort were plotted. The vast majority of subjects are between 18 and 44 and no samples are below 18 years of age.

Supplemental 12: Sex counts by cohort.

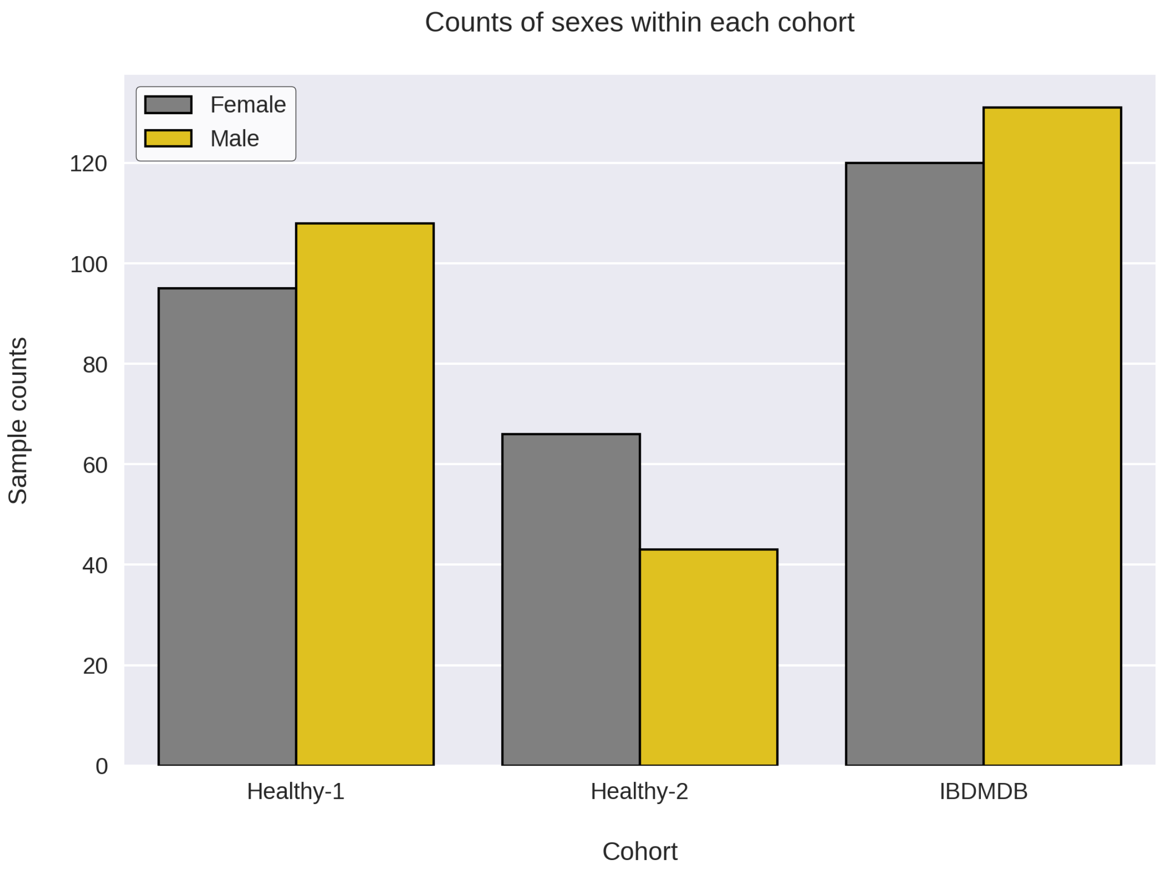

Caption: The counts of samples from a subject of a given sex were plotted for each cohort. There appears to be a greater proportion of females in the Healthy-2 cohort relative, but the IBDMDB and Healthy-1 cohorts appear to have similar proportions of each sex.
